# Generation of virtual monoenergetic images at 40 keV of the upper abdomen and image quality evaluation based on generative adversarial networks

**DOI:** 10.1186/s12880-024-01331-3

**Published:** 2024-06-18

**Authors:** Hua Zhong, Qianwen Huang, Xiaoli Zheng, Yong Wang, Yanan Qian, Xingbiao Chen, Jinan Wang, Shaoyin Duan

**Affiliations:** 1 Department of Radiology, ZhongShan Hospital of Xiamen University, School of Medicine, Xiamen University, Hubinnan Road, Siming District, Xiamen, Fujian 361004 China; 2 Clinical Science, Philips Healthcare, Shanghai, China

**Keywords:** Generative adversarial networks, Upper abdomen, Spectral CT, Virtual monoenergetic images, Conventional images

## Abstract

**Background:**

Abdominal CT scans are vital for diagnosing abdominal diseases but have limitations in tissue analysis and soft tissue detection. Dual-energy CT (DECT) can improve these issues by offering low keV virtual monoenergetic images (VMI), enhancing lesion detection and tissue characterization. However, its cost limits widespread use.

**Purpose:**

To develop a model that converts conventional images (CI) into generative virtual monoenergetic images at 40 keV (Gen-VMI_40keV_) of the upper abdomen CT scan.

**Methods:**

Totally 444 patients who underwent upper abdominal spectral contrast-enhanced CT were enrolled and assigned to the training and validation datasets (7:3). Then, 40-keV portal-vein virtual monoenergetic (VMI_40keV_) and CI, generated from spectral CT scans, served as target and source images. These images were employed to build and train a CI-VMI_40keV_ model. Indexes such as Mean Absolute Error (MAE), Peak Signal-to-Noise Ratio (PSNR), and Structural Similarity (SSIM) were utilized to determine the best generator mode. An additional 198 cases were divided into three test groups, including Group 1 (58 cases with visible abnormalities), Group 2 (40 cases with hepatocellular carcinoma [HCC]) and Group 3 (100 cases from a publicly available HCC dataset). Both subjective and objective evaluations were performed. Comparisons, correlation analyses and Bland-Altman plot analyses were performed.

**Results:**

The 192nd iteration produced the best generator mode (lower MAE and highest PSNR and SSIM). In the Test groups (1 and 2), both VMI_40keV_ and Gen-VMI_40keV_ significantly improved CT values, as well as SNR and CNR, for all organs compared to CI. Significant positive correlations for objective indexes were found between Gen-VMI_40keV_ and VMI_40keV_ in various organs and lesions. Bland-Altman analysis showed that the differences between both imaging types mostly fell within the 95% confidence interval. Pearson’s and Spearman’s correlation coefficients for objective scores between Gen-VMI_40keV_ and VMI_40keV_ in Groups 1 and 2 ranged from 0.645 to 0.980. In Group 3, Gen-VMI_40keV_ yielded significantly higher CT values for HCC (220.5HU vs. 109.1HU) and liver (220.0HU vs. 112.8HU) compared to CI (*p* < 0.01). The CNR for HCC/liver was also significantly higher in Gen-VMI_40keV_ (2.0 vs. 1.2) than in CI (*p* < 0.01). Additionally, Gen-VMI_40keV_ was subjectively evaluated to have a higher image quality compared to CI.

**Conclusion:**

CI-VMI_40keV_ model can generate Gen-VMI_40keV_ from conventional CT scan, closely resembling VMI_40keV_.

## Introduction

Enhanced abdominal computed tomography (CT) is a common diagnostic tool applied for decades in abdominal diseases, including tumors, inflammation, and trauma. However, this method has certain limitations. Firstly, it reflects X-ray attenuation in the body as a whole, making it challenging to analyze tissues in detail, a common issue that arises when attempting to distinguish between calcified plaques and blood infused with iodine [1]. Secondly, its ability to detect soft tissues is limited, especially small, low-contrast soft tissue abnormalities. In certain situation with multiphasic CT yielding a diagnosis with low confidence, additional imaging methods such as MRI or PET-CT may be required. Recently, dual-energy CT (DECT) has emerged as a technology that can potentially reduce the need for additional imaging and improve diagnostic efficiency in multiple disorders [2, 3].

DECT provides additional spectral information that cannot be obtained by conventional CT. This technology improves the sensitivity and accuracy of lesion detection, enables material characterization, and reduces metal artifacts. Therefore, DECT has emerged as a promising diagnostic imaging tool [4, 5]. Such an approach provides notable benefits in terms of suppressing artifacts and enhancing image quality [6, 7]. Compared with conventional contrast-enhanced CT images, virtual monoenergetic images (VMI) at 40–70 keV derived from spectral CT have enhanced contrast and image quality for blood vessels and enhanced tissues. This provides a theoretical and technical basis for optimizing contrast agent injection protocols in enhanced scanning [8]. In lower extremity Computed Tomography Angiography, hepatic portal vein angiography, and contrast-enhanced scanning of the thorax, abdomen, and pelvis, the contrast agent dose can be decreased by 50–65% [9–11]. In individuals with renal insufficiency, ensuring image quality while reducing the contrast agent concentration is essential to prevent potential renal toxicity. Furthermore, factors such as individual variations and circulatory disorders result in suboptimal enhancement of blood vessels and tissues on CT images. Spectral CT allows for retrospective enhancement of the CT value in blood vessels by applying low-energy VMI, thereby improving image quality and enhancing diagnostic accuracy and confidence. This approach eliminates the need for repeated examinations and reduces unnecessary radiation exposure [12]. Multiple studies have demonstrated that VMI_40keV_ exhibits the highest contrast-to-noise ratio, which is advantageous for lesion detection. VMI_40keV_ maximizes the contrast of liver tumors, improves the image quality of multiphase abdominal enhancement scans, and enhances the detection of liver and pancreatic lacerations [13, 14]. However, DECT is more expensive than conventional CT, which limits its widespread adoption. Given this constraint, there is an urgent need to identify a cost-effective alternative that can mimic the advantages of DECT without the substantial financial outlay. This is where the idea of converting CI into VMI_40keV_ becomes critical importance.

Deep Learning is renowned for its reliability, consistency, and accuracy in delivering results. These attributes have led to its extensive application across various domains, particularly in medical imaging [15–18]. Recently, Deep Learning has significantly transformed medical imaging, yielding remarkable advancements in image segmentation, diagnosis, and treatment planning. For examples, ConvUNeXt, a convolutional neural network (CNN) noted for its efficiency in medical image segmentation. Lightweight neural networks, such as those with multiscale feature enhancement, have demonstrated effectiveness in liver CT segmentation [19, 20]. Other notable models like DRU-Net and Dense-PSP-UNet underscore the capabilities of deep CNNs in enhancing both speed and accuracy in medical image segmentation tasks, particularly in liver ultrasound imaging [21, 22]. Additionally, the integration of CNN and transformer architectures, exemplified by CoTr, has further boosted the efficiency of 3D medical image segmentation [23]. Ansari et al. reviewed liver segmentation methods in clinical surgeries over the past decade and proposed a classification based on clinical value to assist clinicians in selecting the most suitable method. They systematically reviewed deep learning-based ultrasound image segmentation techniques over the past five years, summarizing methods, network architectures, loss functions, and the pros and cons of existing approaches for segmenting various organs [24, 25]. Akhtar et al. simulated hepatic resection surgery and assessed the indirect clinical risks of computer-aided diagnostic software, finding that it reduces the time to tumor recurrence compared to manual segmentation [26].

Besides, image generating tasks have attracted increasing attention in the field of computer vision. Among them, Generative Adversarial Network (GAN) models based on CNN, including Pix2Pix-GAN, are commonly used for image-to-image translation and transformation tasks [27]. The Pix2Pix-GAN model achieves image generation by utilizing two neural networks, including a generator and a discriminator. Its architecture typically consists of a U-Net generator, which allows for high-resolution image synthesis, and a patch-based discriminator, which evaluates the generated images at various scales. This design enables the model to focus on both local and global consistency, which is crucial for generating realistic images. Additionally, Pix2Pix GAN employs a loss function that combines adversarial loss, encouraging the generator to produce images indistinguishable from real ones, and content loss, ensuring the generated images retain the content of the input images. This combination of losses helps Pix2Pix-GAN to learn a robust mapping from input to output images, making it a powerful tool for image-to-image translation tasks. Conte et al. applied GAN to generate synthesized missing T1 and FLAIR MRI sequences for a multisequence brain tumor segmentation model [28]. Kawahara et al. employed GAN to generate monoenergetic CT images in DECT from kilovoltage CT scans, concluding that the proposed model offers a viable alternative for reconstructing monoenergetic CT images in DECT from single-energy CT scans [29].

In this study, we developed a CI-VMI_40keV_ model based on Pix2pix-GAN.This model had been enhanced by augmenting the depth of its generator network, enabling it to generate Gen-VMI_40keV_ from CI acquired from upper abdominal CT scans. We then assess its performance by conducting a comparative analysis of image quality among Gen-VMI_40keV_, VMI_40keV_, and CI. The key contributions of this study are:

- 3D original image that has been segmented into a series of 2D images, each with a size of 512 × 512. No cropping or resampling was performed during this transformation, and the metadata was preserved to allow for corresponding region of interest (ROI) delineation for subsequent image quality assessment.
- The best generator model was selected using a validation dataset based on Mean Absolute Error (MAE), Peak Signal-to-Noise Ratio (PSNR), and Structural Similarity (SSIM).
- The quality of Gen-VMI_40keV_ was evaluated using three sets of test groups. The first two groups were compared against corresponding images from our center, while the third group consisted of external data.
- An encoding and decoding layer were added to accommodate 512 × 512 data and optimize the performance of the generator model.
- Image quality was assessed using a combination of objective and subjective evaluation methods.

The remainder of this paper is structured as follows. Section 2 delves into the nuances of dataset preparation, the characteristics of patient datasets, the Pix2Pix framework with its architectural components and associated parameters, and the models employed. Furthermore, it discusses the model’s evaluation through objective indices of image quality, as well as both objective and subjective assessments of image quality. Section 3 presents the details of results and image quality analysis. Section 4 contains a discussion of the paper, concludes the paper’s value, acknowledging the study’s limitations and outlining potential avenues for future research.

## Materials and methods

This retrospective study was approved by the Ethics Committee of Zhongshan Hospital affiliated to Xiamen University (IRB approval number: XMZSYY-AF-SC-12-03), who waived the requirement for informed consent.

### Patient datasets

The study included training and validation sets, as well as two test groups (Group 1 and 2) of patients administered three-phase contrast-enhanced spectral CT scans of the upper abdomen. Additionally, another test group (Group 3) of patients who underwent conventional CT was included. The inclusion criterion for the study was the availability of portal venous phase CT images. Exclusion criteria were: (1) poor CT image quality with severe motion artifacts; (2) metallic implants causing significant radiographic artifacts; additional exclusions criterion for Test group 2&3: (3) unclear lesion appearance in the portal venous phase; (4) lesions occupying the liver, thus difficulty in distinguishing the normal tissue.

**Fig. 1:**
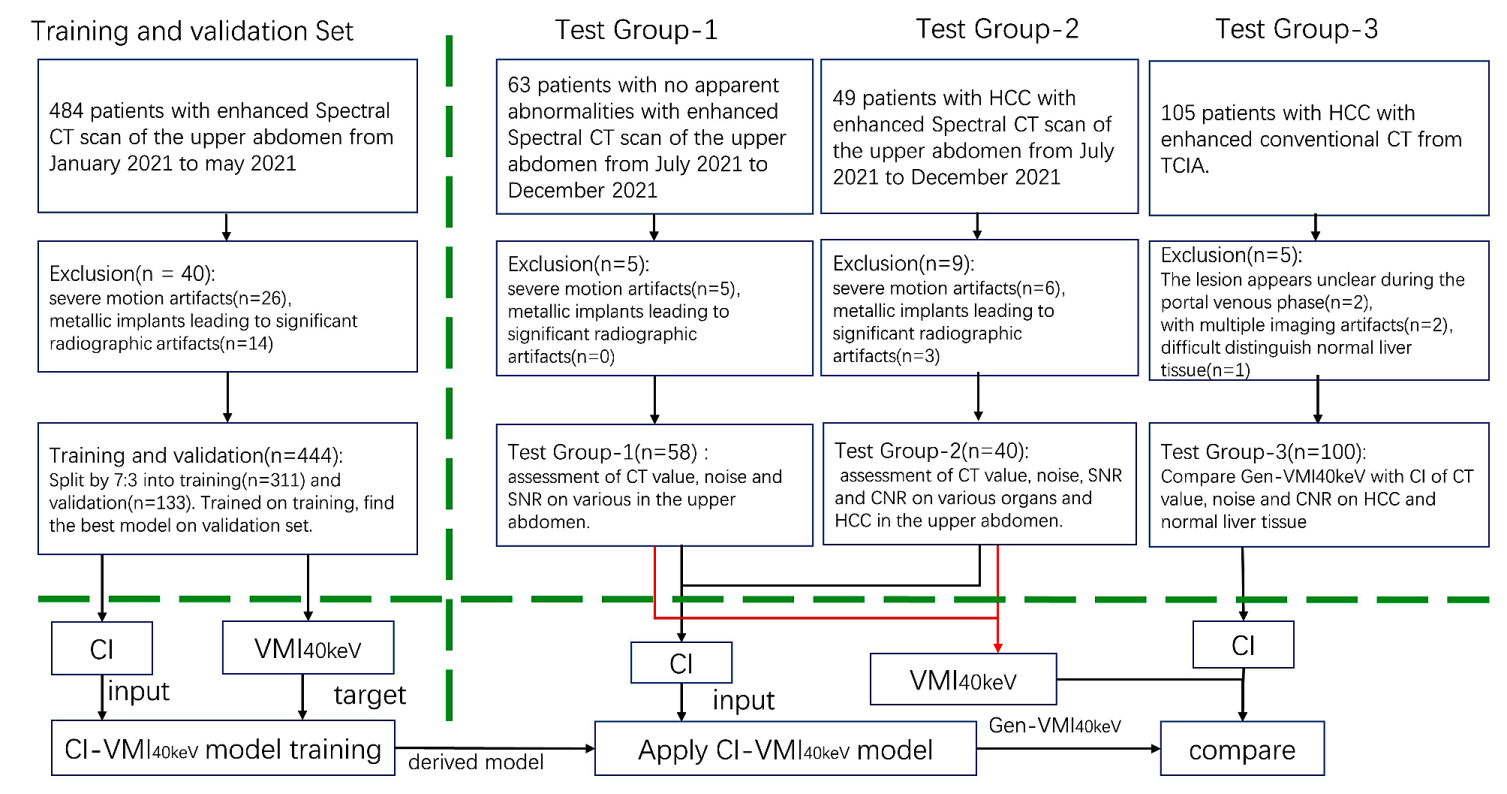
Flowchart of the training and validation sets and three test groups

The training and validation sets (*n* = 444) were randomly selected from January 2021 to May 2021 from Zhongshan hospital affiliated to Xiamen University. Test group 1 (58 cases with no apparent abnormalities) and Test group 2 (40 cases with HCC) were randomly selected from July 2021 to December 2021 using Python (version 3.8) based on imaging report, respectively. Test group 3 patients were obtained from The Cancer Imaging Archive (TCIA, https://www.cancerimagingarchive.net/) [30]. Figure 1 depicts the process of obtaining datasets, including the application of exclusion criteria, as well as the stages of training, validation, and tests.

### Image acquisition

All the examined patients underwent three-phase contrast-enhanced CT using a dual-layer spectral detector CT (IQon, Philips, The Netherlands). Patients fasted for 5–7 h, drank 600 ml of water each, and were positioned supine with raised arms. Scans were performed from the upper liver to above the umbilicus. The main scanning parameters were: collimation, 0.625 mm × 64; pitch, 1.2 and 0.75 s per rotation; field-of-view (FOV), 35 cm; tube voltage, 120 kVp; automatic tube current modulation (90–180 mAs). The reconstructed images had a matrix of 512 × 512 and a slice thickness of 1.0 mm. After a non-contrast scan, patients received 60–75 ml of a iodinated contrast agent (Optiray, 300 mg/ml, Bayer) via a power injector at 3.0-3.2 ml/s. Arterial and portal venous phase scans occurred at 25 and 60 s after injection.

Finally, two-phase enhanced data were reconstructed using projection space spectral reconstruction to generate Spectral Based Images (SBI). The obtained portal venous SBI were transferred to a dedicated workstation (IntelliSpace V9, Philips Healthcare) for further analysis. VMI_40keV_ and CI were derived from SBI with a slice thickness of 3 mm.

### Characteristics of patient datasets

Table 1 summarizes baseline patient data in the training and validation sets and the three test groups, respectively. The final training set from our institution included 311 patients, aged 52.2 ± 14.1 years, including 141females, while the final validation set comprised 133 patients (aged 53.2 ± 15.2 years, with 61 females). The final numbers of patients in Test groups 1 (no apparent abnormalities) and 2 (HCC) from our institution were 58 (aged 48.4 ± 16.5 years, including 27 females) and 40 (aged 61.0 ± 13.5 years, including 6 females), respectively. The Test group 3 included 100 patients from TCIA, whose characteristics were unknown.

**Table 1:** Characteristics of patient data sets

Item	Training (*n* = 311)	Validation (*n* = 133)	Test group1 (*n* = 58)	Test group2 (*n* = 40)	Test group3 (*n* = 100)
Female/male/n	141/170	61/72	27/31	6/34	Unknown
Age/year	52.2 ± 14.1	53.2 ± 15.2	48.4 ± 16.5	61.0 ± 13.5	Unknown
Scanner used	IQon	IQon	IQon	IQon	Unknown
No. of CI	21,396	9709	3209	2886	9049
No. of VMI_40keV_	21,396	9709	3209	2886	
Pathogeny	All	All	Normal	HCC	HCC

a. HCC = Hepatocellular Carcinoma, VMI = virtual monoenergetic images, CI = conventional images

b. All mean the data include no apparent abnormalities, HCC, hepatic cyst, hepatic hemangioma, hepatic metastases, stomach cancer and so on

### Training the CI-VMI_40keV_ model to generate Gen-VMI_40keV_ from CI

**Fig. 2:**
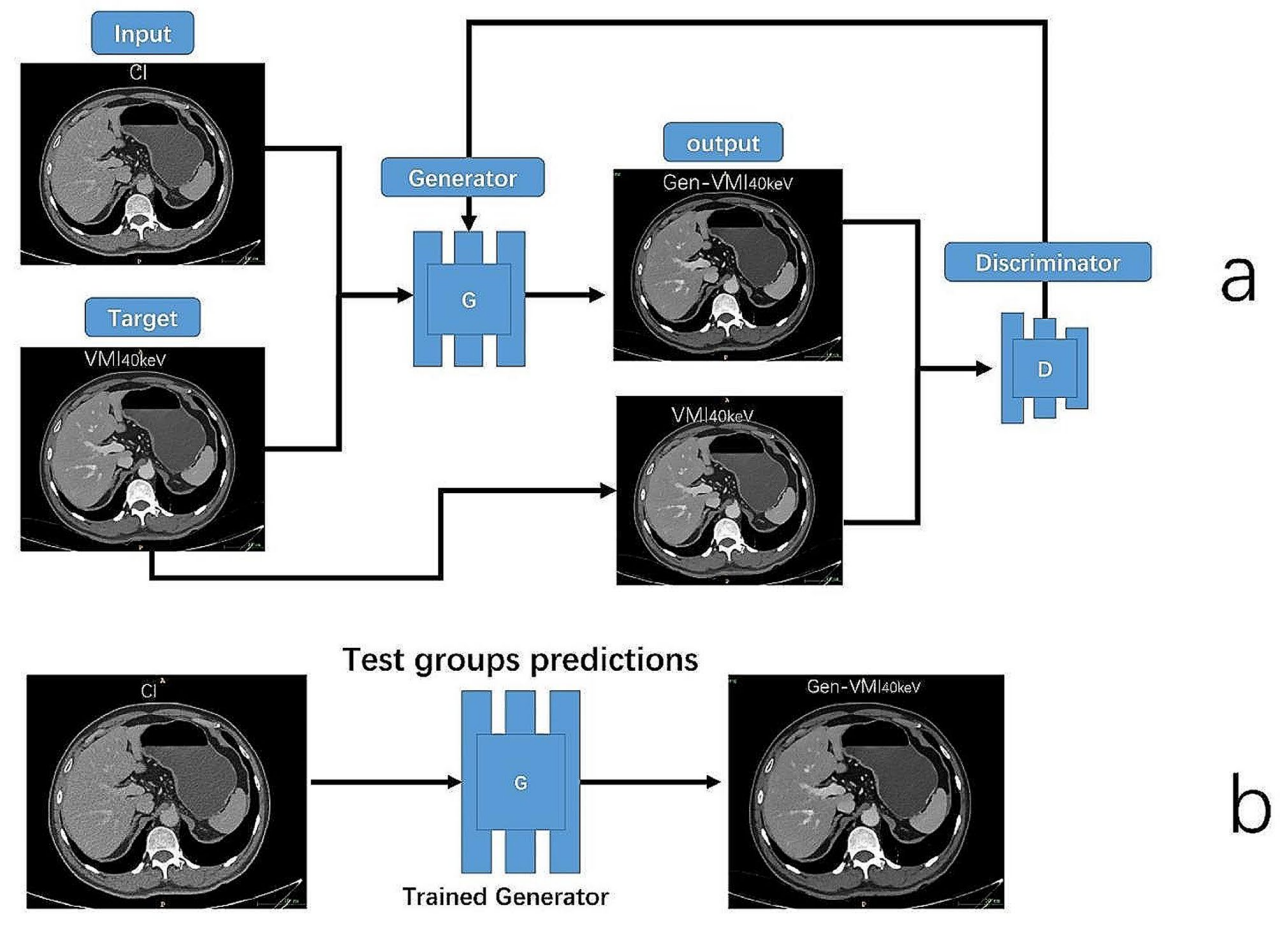
(**a**) Schematic diagram of the CI-VMI_40keV_ model. (**b**) Synthesis Gen-VMI_40keV_ upper abdominal CT images from conventional images

We utilized the Pix2Pix framework, a conditional GAN designed for image-to-image translation, to train the GAN model to generate Gen-VMI_40keV_ from CI. Prior to training, CT intensities ranging from − 1024 to 3071 were normalized to the (-1, 1) range. During the training process, this model was provided with paired sections, with one pair section belonging to the source (CI) and target (VMI_40keV_) domains. By leveraging the adversarial training, the GAN model learned to generate realistic VMI_40keV_, referred to as Gen-VMI_40keV_. The details of the CI-VMI_40keV_ model training and Gen-VMI_40keV_ from CI are shown in Fig. 2.

The CI-VMI_40keV_ model in this study was trained for 250 epochs, as described by Isola et al. [27]. The training process involved alternating between training the discriminator and the generator for a gradient descent step. To ensure a balanced training, the discriminator loss was halved to slow down its training compared with the generator. The final loss function consisted of a combination of BCEWithLogitsLoss and L1 loss. During training, mini-batch random gradient descent was used with a batch size of 32. The Adam optimizer was utilized with a learning rate of 0.0002 and momentum parameters set to b1 = 0.5 and b2 = 0.999. These settings were crucial for optimizing the model’s performance and achieving the desired image translation results.

The Python software version 3.8 (Python Software Foundation) and PyTorch (version 1.12.1, https://pytorch.org/) were utilized. Model training and predictions were performed on a Linux workstation running Ubuntu version 20.04, equipped with an NVIDIA GeForce GTX 3090 GPU with 24 GB memory (NVIDIA, Santa Clara, CA, USA).

### Model evaluation with objective indexes of image quality

To evaluate the performances of the models and select the best generative model, CI from the validation set were used as input to the CI-VMI_40keV_ model. MAE, Peak PSNR, and SSIM were used for model assessment. They were derived as follows:

1 $$\text{M}\text{A}\text{E}(\text{I},\text{K}) =\frac{1}{\text{n}}\sum _{\text{i}=1}^{\text{n}}\left(\left|\text{I}-\text{K}\right|\right)$$

2 $$\text{M}\text{S}\text{E}(\text{I},\text{K}) =\frac{1}{\text{n}}{\sum _{\text{i}=1}^{\text{n}}(I-K)}^{2}$$

3 $$\text{P}\text{S}\text{N}\text{R} = 10 \times {\text{log}}_{10}\left(\frac{{\left({2}^{\text{n}}-1\right)}^{2}}{\text{M}\text{S}\text{E}}\right)$$

4 $$\text{S}\text{S}\text{I}\text{M}\left(\text{I},\text{K}\right)=\frac{\left(2{\mathcal{U}}_{\text{I}}{\mathcal{U}}_{\text{K}}+{\text{C}}_{1}\right)\left(2{{\sigma }}_{\text{I}\text{K}}+{\text{C}}_{2}\right)}{\left({{\mathcal{U}}_{\text{I}}}^{2}+{{\mathcal{U}}_{\text{K}}}^{2}+{\text{C}}_{1}\right)\left({{{\sigma }}_{\text{I}}}^{2}+{{{\sigma }}_{\text{K}}}^{2}+{\text{C}}_{2}\right)}$$

MAE is the average absolute difference between the generated (I) and actual (K) images. The closer the MAE to 0, the closer the Gen-VMI_40keV_ to VMI_40keV_. PSNR assesses the noise distribution difference between Gen-VMI_40keV_ and VMI_40keV_, where n represents the number of bits for pixel representation; MSE is the mean squared difference between I and K. A PSNR value of 20 ∼ 30dB indicates poor image quality; 30 ∼ 40dB implies noticeable image distortion but acceptable quality, and > 40dB suggests extremely high image quality. SSIM is a full-reference image quality assessment metric. In this context, ${\mathcal{U}}_{\text{I}}$ is the mean of I, ${\mathcal{U}}_{\text{K}}$ represents the mean of K, ${{{\sigma }}_{\text{I}}}^{2}$ is the variance of I, ${{{\sigma }}_{\text{K}}}^{2}$ denotes the variance of K, and ${{\sigma }}_{\text{I}\text{K}}$ represents the covariance between I and K, c_1_ and c_2_ are constants utilized to uphold stability, where c_1_ = (k_1_L)^2^ and c_2_ = (k_2_L)^2^. Here, k_1_ = 0.01 and k_2_ = 0.03. L symbolizes the dynamic range of pixel values, typically set to L = 255. The SSIM value ranges from 0 to 1, with a larger value indicating low image distortion.

The best generative model was used to generate Gen-VMI_40keV_, and the CT values of the Gen-VMI_40keV_ were restored to the range of -1024 to 3071 HU. The coordinates and spacing of the obtained CI were assigned to the Gen-VMI_40keV_, so that Gen-VMI_40keV_, CI, and VMI_40keV_ had the same spacing and spatial coordinates.

### Objective evaluation of image quality

The objective evaluation was performed by a physician with seven years of experience in abdominal imaging. Using the medical image segmentation software ITK-Snap on the Test group 1, the regions of interest (ROIs) were delineated on CI. The ROIs were placed in the following areas, including 8 Couinaud segments of the liver, head/body/tail of the pancreas, spleen, subcutaneous adipose tissue, abdominal aorta, and erector spinae muscle. The CT value (mean) and the corresponding standard deviation (SD) were obtained. The areas of ROIs ranged from 100 to 1000 mm² and avoided blood vessels while maintaining density uniformity. Then, the ROIs were applied to VMI_40keV_ and Gen-VMI_40keV_, ensuring consistent ROI sizes across images, and measurements were performed thrice to obtain an average value. The SD was considered the noise value, and the signal-to-noise ratio (SNR) was determined for each group of ROIs in the three image types as SNR = CT/SD. In Test groups 2 and 3, the same approach was applied to place ROIs in both HCC and normal liver tissues. The CT value (mean) and the corresponding SD were determined. The areas of ROIs ranged from 30 to 1000 mm², avoiding necrosis, blood vessels, calcification, etc. The contrast-to-noise ratio (CNR) for liver cancer was assessed as CNR = (CT_HCC_ – CT _liver tissue_) / SD _liver tissue_.

### Subjective evaluation of image quality

Two physicians each with 7 years of experience in abdominal imaging performed subjective ratings for image quality on Test groups 1, 2, and 3. In case of any discrepancy, a third senior physician with 15 years of experience made the final determination for subsequent analysis. The scoring was performed with a Likert 5-point scale as follows: 1, unidentifiable anatomical structures, extremely severe noise, very high image granularity, and poor image quality; 2, difficult anatomical structures to discern, blurry edges, severe noise, high image granularity, and relatively poor image quality; 3, some unclear anatomical structures, somewhat blurry edges, moderate noise, relatively high image granularity, and fair image quality; 4, quite clear anatomical structures, easily identifiable edges, minimal noise, small image granularity, and good image quality; 5, clear anatomical structures, smooth and clear edges, no apparent noise, minimal image granularity, and excellent image quality.

### Statistical analysis

Statistical analysis was performed with R (version 4.1.0, https://www.r-project.org/), and statistical significance was defined as two-sided *P* < 0.05. The Kolmogorov-Smirnov test was used to assess the normality of continuous variables. Normally distributed data were expressed as mean ± standard deviation (SD), and non-normally distributed data as median (interquartile range) [M (Q1, Q3)]. In Test groups 1 and 2, both quantitative and quantitative indexes derived from CI, VMI_40keV_ and Gen-VMI_40keV_ were compared by the Friedman test. In case of statistically significant difference, post-hoc pairwise comparisons were performed by Dunn- Bonferroni correction. Pearson’s and Sperman’s correlation analyses were used to examine the correlations of CT values, noise, SNR, and CNR between VMI_40keV_ and Gen-VMI_40keV_. The agreement of quantitative measurements from VMI_40keV_ and Gen-VMI_40keV_ was assessed with Bland-Altman plots. In the Test group 3, the Wilcoxon signed-rank test was applied to compare quantitative measurements and quantitative indexes from CI and Gen-VMI_40keV_.

## Results

### Selection of the best generator model

**Fig. 3:**
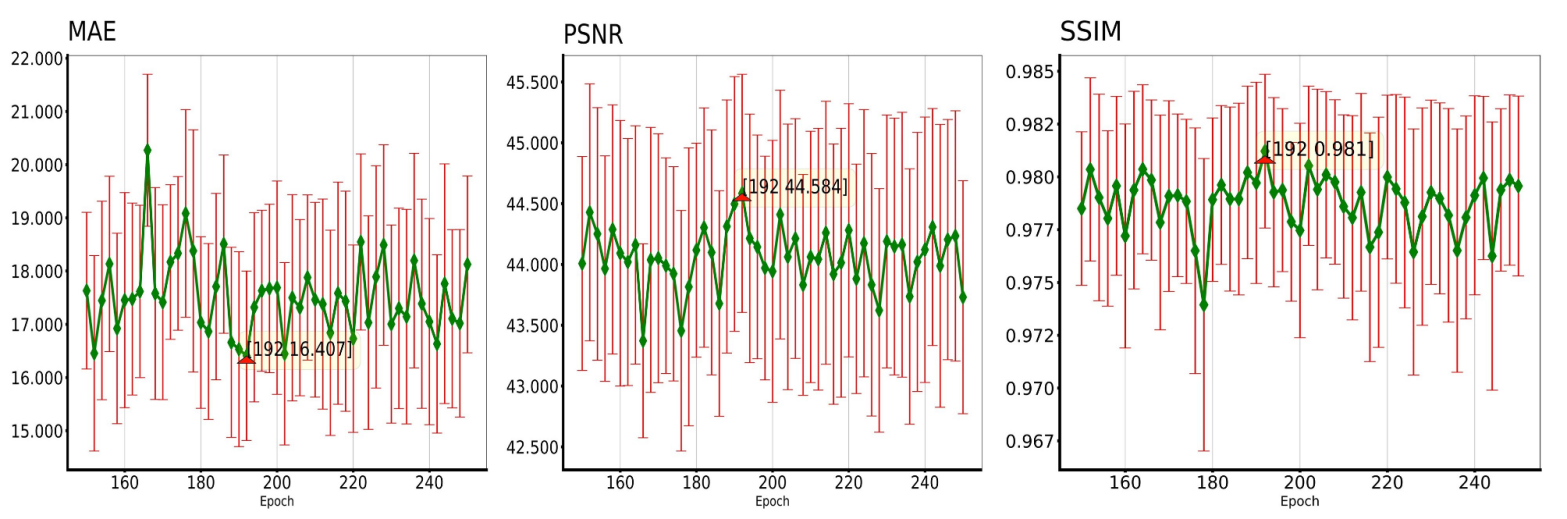
Presents the mean absolute error (MAE), peak signal-to-noise ratio (PSNR), and structural similarity index measure (SSIM) between VMI_40keV_ and Gen-VMI_40keV_ across different epochs ranging from 150 to 250 in the validation set. The line graphs depict the mean, and each point is accompanied by an error bar. The lower and upper whiskers on the vertical line represent the mean minus the standard deviation and mean plus the standard deviation, respectively

In the validation set, all PSNR and SSIM values were above 40 and 0.96, respectively. Figure 3 shows that the generator model at step 192 was selected as the best model, with the lowest MAE (16.407), highest PSNR (44.584), and highest SSIM (0.981).

### Objective evaluation of images

Except for the noise difference in the erector spinae muscle in the Test group 1, there were significant differences in CT value, noise, SNR, and CNR among the various groups for liver, pancreas, spleen, subcutaneous fat, aorta, and erector spinae muscle. Additionally, Gen-VMI_40keV_ and VMI_40keV_ exhibited significant positive correlations in CT value, noise, SNR, and CNR for various organs and HCC (all *P* < 0.01). The Pearson’s and Spearman’s correlation coefficients ranged from 0.645 to 0.980 (Table 2).

**Table 2:** Analysis of CT-value, noise SNR and CNR between CI, VMI40keV and Gen-VMI40keV in Test group 1 and Test group 2

Indicators	No. of Patients	Organs	CI	VMI_40kev_ VMI_40kev_	Gen-VMI_40kev_	χ²	PCC&SCC
CT value (HU)	58	Liver	99.5 ± 14.5	192.0 ± 45.2^#^	196.4 ± 31.1^#^	90.448*	0.955* (0.920,0.976)
	58	Pancreas	80.3(73.6,84.7)	160.7(141.5, 179.8)^#^	169.4(155.0,185.7)^#^	96.966*	0.926* (0.853,0.961)
	58	Spleen	98.8 ± 12.8	216.5 ± 42.0^#^	222.9 ± 33.2^#^	90.448*	0.980* (0.964,0.990)
	58	fat	-110.0(-113.6, -107.6)	-179.9(-185.1, -175.4)^#^	-179.0(-183.9, -175.1)^#^	89.207*	0.939* (0.861,0.973)
	58	Aorta	123.4 ± 19.1	317.9 ± 63.0^#^	320.6 ± 54.1^#^	89.793*	0.969* (0.948,0.982)
	58	Muscle	58.2 ± 4.6	75.5 ± 10.3^#^	75.7 ± 9.7^#^	87.034*	0.822* (0.701,0.907)
Noise (HU)	58	Liver	19.1 ± 2.0	21.4(19.4, 22.4)^#^	21.1 ± 2.7^#^	79.276*	0.826* (0.703,0.896)
	58	Pancreas	22.1(20.9,24.3)	25.8(23.0, 28.2)^#^	27.2(24.8,29.1)^#^	81.827*	0.806* (0.676,0.877)
	58	Spleen	19.5(18.5,20.4)	21.6 ± 3.3^#^	21.1(19.2,23.3)^#^	55.276*	0.891* (0.812,0.928)
	58	Fat	15.6 ± 1.6	16.7 ± 2.2^#^	15.8 ± 1.9	47.697*	0.959* (0.932,0.977)
	58	Aorta	22.4(21.1,23.8)	22.9 ± 3.8	26.4(24.6, 29.1)^#^	81.345*	0.673* (0.504,0.801)
	58	Muscle	19.9 ± 2.2	20.4 ± 3.3	20.0 ± 2.9	2.675(*p* = 0.262)	0.923* (0.849,0.966)
SNR	58	Liver	5.3 ± 1.1	9.2 ± 2.8^#^	9.5 ± 2.1^#^	89.207*	0.899* (0.801,0.952)
	58	Pancreas	3.7 ± 0.7	6.4 ± 1.4^#^	6.4 ± 1.2^#^	87.000*	0.873* (0.792,0.926)
	58	Spleen	5.1 ± 0.9	10.2 ± 2.1^#^	10.5 ± 1.7^#^	91.172*	0.938* (0.902,0.963)
	58	fat	-7.1 ± 1.0	-10.9 ± 1.8^#^	-11.5 ± 1.7^#^	106.862*	0.971* (0.949,0.985)
	58	Aorta	5.5 ± 1.3	14.4 ± 4.1^#^	12.2 ± 3.2^#^	108.552*	0.877* (0.764,0.939)
	58	Muscle	3.0 ± 0.5	3.7(3.3,4.1)^#^	3.9 ± 0.9^#^	79.818*	0.843* (0.705,0.933)
CT value (HU)	40	HCC	68.2 ± 18.6	133.8 ± 51.1^#^	135.6 ± 40.9^#^	60.250*	0.970*(0.953,0.982)
	40	Liver	100.6 ± 11.1	204.5 ± 33.5^#^	203.9 ± 24.9^#^	60.050*	0.926*(0.869,0.960)
Noise (HU)	40	HCC	21.1 ± 2.7	26.6(22.8, 31.8)^#^	26.4 ± 3.8^#^	48.600*	0.684*(0.481,0.827)
	40	Liver	18.5 ± 2.3	19.6(18.2, 21.5)^#^	19.9(18.1,22.1)^#^	30.050*	0.645*(0.412,0.801)
CNR	40	HCC/Liver	1.6(1.1,2.2)	3.3(2.2,4.8)^#^	3.4 ± 2.0^#^	41.150*	0.938*(0.851,0.971)

a. fat means subcutaneous fat. VMI = virtual monoenergetic images. CI = conventional images. SNR = signal-to-noise ratio

CNR = contrast-to-noise ratio. HCC = hepatocellular carcinoma. PCC = Pearson correlation coefficient(VMI40kev vs. Gen-VMI40kev); SCC = Spearman correlation coefficient(VMI40kev vs. Gen-VMI40kev). When the data follows a normal distribution, the Pearson correlation coefficient is used for Correlation testing. When the data does not follow a normal distribution, the Spearman correlation coefficient is applied for Correlation testing

b. # means the difference compared to CI are statistically significant

c. * = Correlation is significant (P-value < 0.05, 2-tailed)

Intra-group comparisons of CT values, SNR and CNR showed that both Gen-VMI_40keV_ and VMI_40keV_ had significantly higher CT values than CI (all *P* < 0.01). In the intra-group comparison of noise, except for subcutaneous fat and erector spinae muscle in Gen-VMI_40kev_ vs. CI, and aorta and erector spinae muscle in VMI_40kev_ vs. CI, there were no statistically significant differences in noise. For all other organs, Gen-VMI_40kev_ and VMI_40kev_ had slightly higher noise compared with CI, with statistically significant differences (*P* < 0.01).

In the Test group 3, there were statistically significant differences in CT value, noise, and CNR between HCC and normal liver parenchyma for Gen-VMI_40keV_ versus.

CI (*P* < 0.01) (Table 3).

**Table 3:** Comparison of CT-value, noise and CNR between CI and Gen-VMI40keV in Test group 3

Indicators	No. of patients	Organs	CI	Gen-VMI_40keV_	*P*-value
CT-value	100	HCC	109.1(88.2,126.2)	220.5(170.6,258.6)	< 0.01
CT-value	100	Liver	112.8(98.2,134.1)	220.0(195.0,266.1)	< 0.01
Noise	100	HCC	21.6 ± 5.7	27.5(21.1,34.8)	< 0.01
Noise	100	Liver	18.3(15.2,22.5)	29.8 ± 12.5	< 0.01
CNR	100	HCC/Liver	1.2(0.6,1.7)	2.0(1.1,3.4)	< 0.01

a. VMI = virtual monoenergetic images. CI = conventional images

b. HCC = hepatocellular carcinoma. CNR = contrast-to-noise ratio

**Fig. 4:**
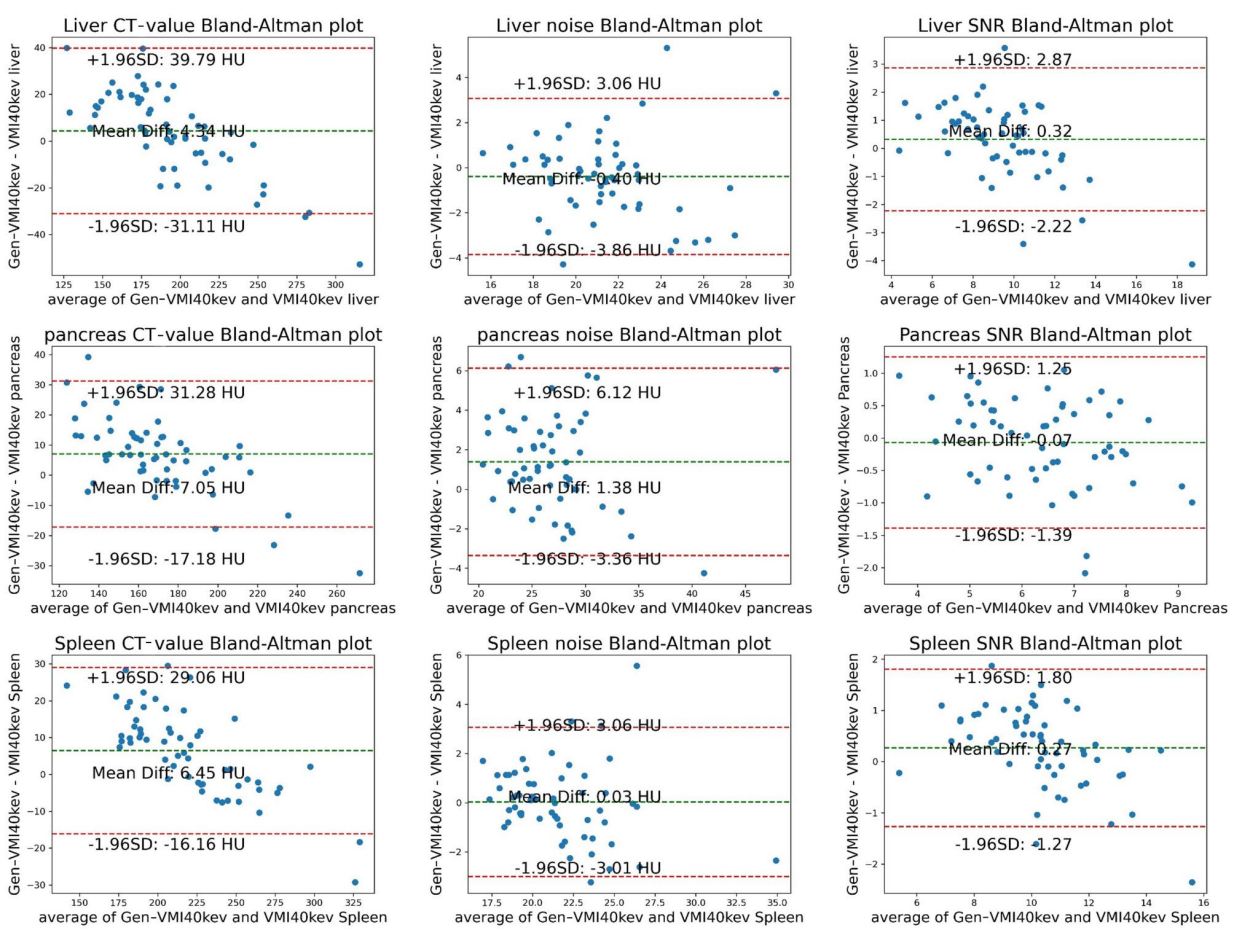
Bland-Altman plot showing VMI_40keV_ (CT-value, noise and SNR) and Gen-VMI_40keV_ (CT-value, noise and SNR) on the liver, pancreas and spleen in the Test group 1. The middle horizontal line represents the mean value of the difference between VMI_40keV_ and Gen-VMI_40keV_. The difference between the upper and lower horizontal lines represents the 95% confidence interval

**Fig. 5:**
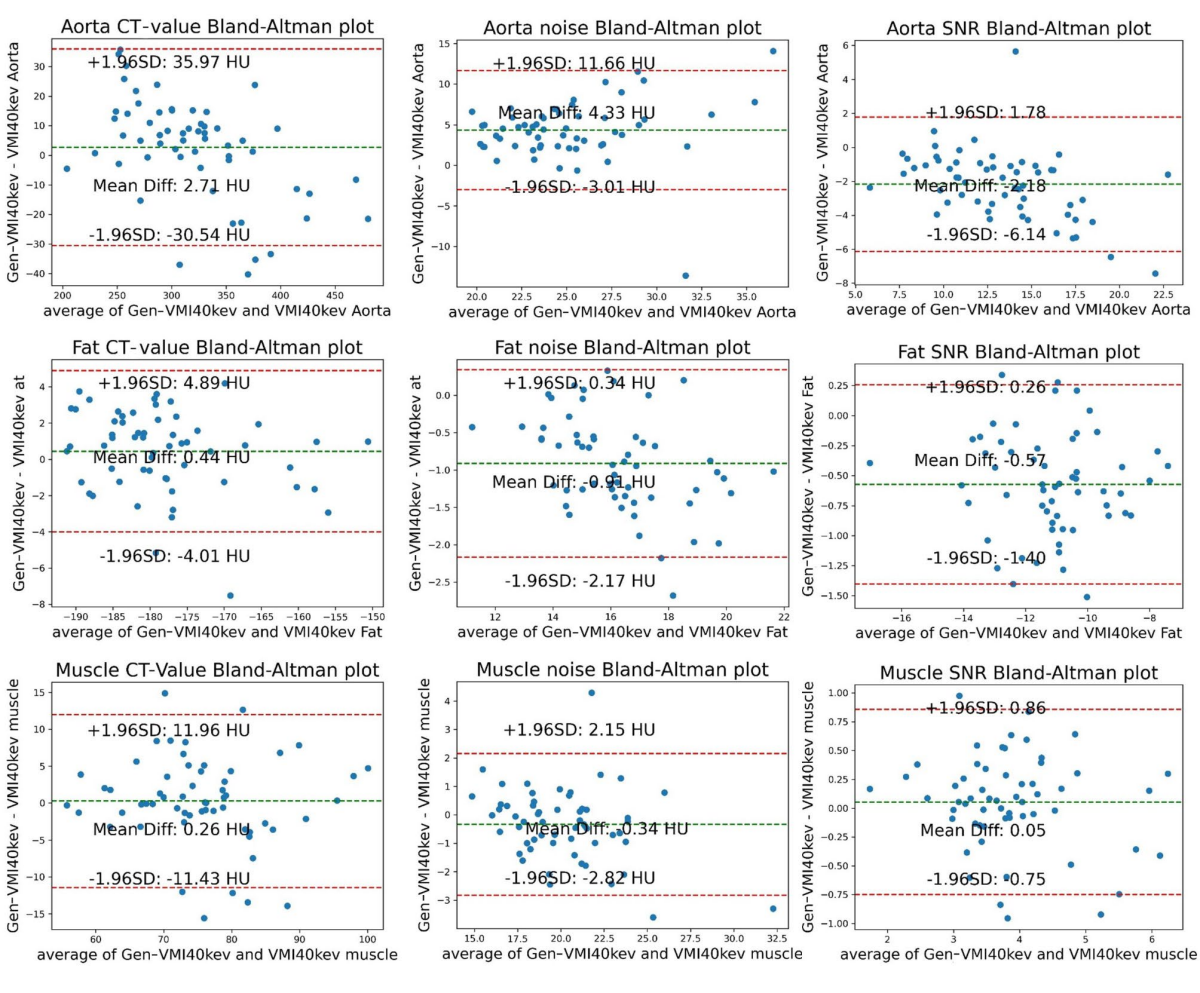
Bland-Altman plot showing VMI_40keV_ (CT-value, noise and SNR) and Gen-VMI_40keV_ (CT-value, noise and SNR) on the aorta, subcutaneous fat and muscle in the Test group 1. The middle horizontal line represents the mean value of the difference between VMI_40keV_ and Gen-VMI_40keV_. The difference between the upper and lower horizontal lines represents the 95% confidence interval

Bland-Altman plots for the Test group 1 showed mean differences in CT value for the liver, pancreas, spleen, subcutaneous fat, aorta, and erector spinae muscle between Gen-VMI_40keV_ and VMI_40kev_ of 4.34 HU, 7.05 HU, 6.45 HU, 0.44 HU, 2.71 HU, and 0.26 HU, respectively. Mean differences in noise were − 0.40 HU, 1.38 HU, 0.03 HU, -0.91 HU, 4.33 HU, and − 0.34 HU, respectively. Mean differences in SNR were 0.32, -0.07, 0.27, -0.57, -2.18, and 0.05, respectively. These measurement data were mostly within the respective 95% confidence intervals (Figs. 4 and 5).

**Fig. 6:**
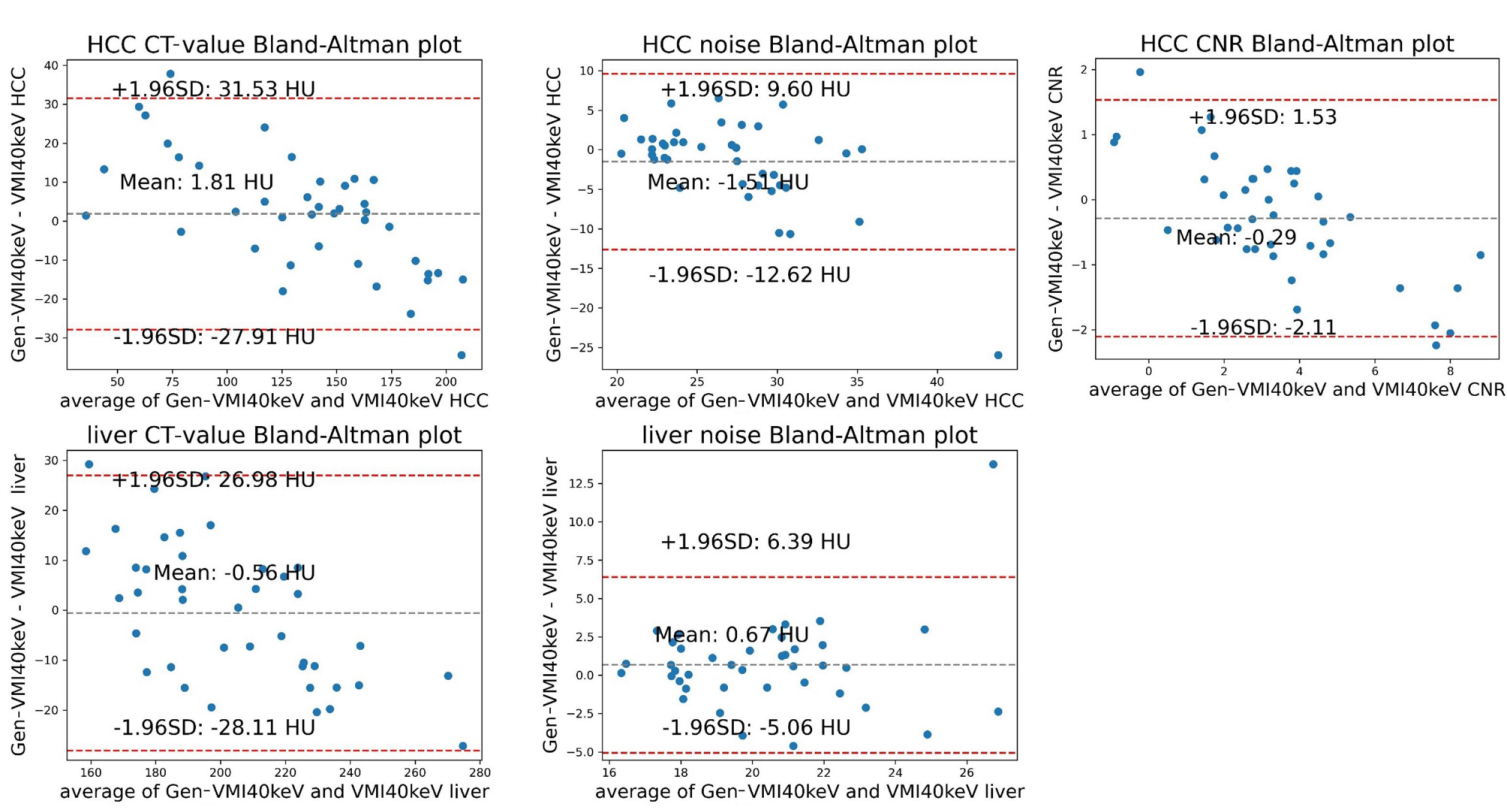
Bland-Altman plot showing VMI_40keV_ (CT-value, noise and CNR) and Gen-VMI_40keV_ (CT-value, noise and CNR) on the HCC, liver in the Test group 2. The middle horizontal line represents the mean value of the difference between VMI_40keV_ and Gen-VMI_40keV_. The difference between the upper and lower horizontal line represents a 95% confidence interval

Bland-Altman plots for the Test group 2 showed mean differences in CT values between HCC and normal liver parenchyma for Gen-VMI_40keV_ and VMI_40keV_ of 1.81 HU and − 0.56 HU, respectively. Mean differences in noise were − 1.51 HU and 0.67 HU, respectively. The mean difference in CNR was − 0.29. Most of these measurement data were within the respective 95% confidence intervals (Fig. 6).

### Subjective evaluation of images

In Test groups 1 and 2, median subjective image quality ratings for CI, Gen-VMI_40keV_, and VMI_40keV_ were 4, 5, and 5, respectively. The overall differences among groups were statistically significant (*P* < 0.01). Intra-group comparisons revealed that both Gen-VMI_40keV_ and VMI_40keV_ had significantly higher image quality compared with CI (*P* < 0.01), with no statistically significant difference between Gen-VMI_40keV_ and VMI_40keV_. In the Test group 3, statistically significant differences (*P* < 0.01) were found in image quality indexes between Gen-VMI_40keV_ and CI (Table 4**)**.

**Table 4:** Comparison of subjective scoring of CI, VMI40keV and Gen-VMI40keV in Test group-1, 2&3[M(Q1, Q3)]

Group		Image Type						*P* (VMI_40keV_VS Gen-VMI40keV)
		CI	VMI_40kev_	Gen-VMI_40kev_	χ^2^	Z cores	*P*	
Test 1	Image quality	4(4,4)	5(5,5)^#^	5(5,5)^#^	115.349		<0.01	0.926
Test 2	Image quality	4(4,4)	5(5,5)^#^	5(5,5)^#^	76.934		<0.01	0.696
Test 3	Image quality	5(4,5)		5(5,5)^#^		-4.849	<0.01	

a. VMI = virtual monoenergetic images. CI = conventional images

b. M(Q1, Q3): Median (Lower Quartile, upper Quartile)

c. ^#^ = the difference compared to CI are statistically significant

## Discussion

In recent years, it was demonstrated that jointly training of the generator and discriminator may improve tasks such as image synthesis and cross-mode image transformation in medical imaging [31–33]. The present study confirms the feasibility of medical image synthesis. In this study, Gen-VMI_40keV_ generated from CI by CI-VMI_40keV_ model were similar to VMI_40keV_ acquired from DECT. This corroborates Yoshinori Funama et al., who conducted a similar study on generating pseudo low-monoenergetic CT images of the abdomen from 120-kVp CT images using cGAN [34]. MAE, PSNR, and SSIM were employed to compare Gen-VMI_40keV_ and VMI_40keV_ in this study. These three metrics are commonly used for image quality assessment in the field of image processing, and may help measure the similarity between Gen-VMI_40keV_ and VMI_40keV_, with SSIM showing a correlation with the perceived quality within the context of the human visual system [35–37]. The results revealed that all models achieved PSNR and SSIM values above 40 and 0.98, respectively, in the validation dataset. This indicates that the models, after a certain number of training steps, produced Gen-VMI_40keV_ in the validation dataset with a high degree of similarity to VMI_40keV_ in terms of CT value, noise distribution, and anatomical structure. Subsequently, we selected the step model from all models reaching the 192nd step, with the lowest MAE and the highest PSNR and SSIM values, for further evaluation in the test groups. In this study, the PSNR and SSIM results we have achieved demonstrate a superior performance level. This discrepancy may be attributed to our utilization of an image resolution of 512*512 and an augmentation in the depth of the generator network in CI-VMI_40keV_ model to accommodate images with a resolution of 512*512. Compared with previous reports [29, 34], the current model was further validated using external test groups (with and without lesions) and CT values were compared between Gen-VMI_40keV_ and original VMI_40keV_ generated from a spectral CT scanner.

Dual-layer detector spectral CT significantly improves the contrast of enhanced tissues in VMI_40keV_, with image noise at a lower level. This further leverages the advantages of low-energy VMI in lesion visualization and detection [38, 39]. Table 2 show that compared to CI, both VMI_40keV_ and Gen-VMI_40keV_ significantly improved CT values for all organs, as well as SNR and CNR, indicating an enhancement in image quality. Notably, despite the significant increase in CT values, the noise in Gen-VMI_40keV_ was similar to or slightly higher than that of CI, demonstrating the model’s robustness to noise. The CT values, noise, SNR, and CNR between VMI_40keV_ and Gen-VMI_40keV_ were highly correlated, with correlation coefficients ranging from 0.645 to 0.980, indicating that Gen-VMI_40keV_ well preserved the advantages of VMI_40keV_. Table 3 validates the model’s performance in an external test group, where Gen-VMI_40keV_ continues to exhibit significant improvements in CT values and CNR compared to CI. The current findings suggest that Gen-VMI_40keV_, similar to VMI_40keV_, offers the increased SNR and CNR, resulting in the improved visualization of abdominal organs and HCC lesions. This improvement holds true even when working with images from scanners of different manufacturers. Gen-VMI_40keV_ effectively enhances contrast between abnormal lesions and background tissues, raises vascular enhancement CT values, improves image quality, increases the detection rate for small lesions, and boosts diagnostic confidence.

**Fig. 7:**
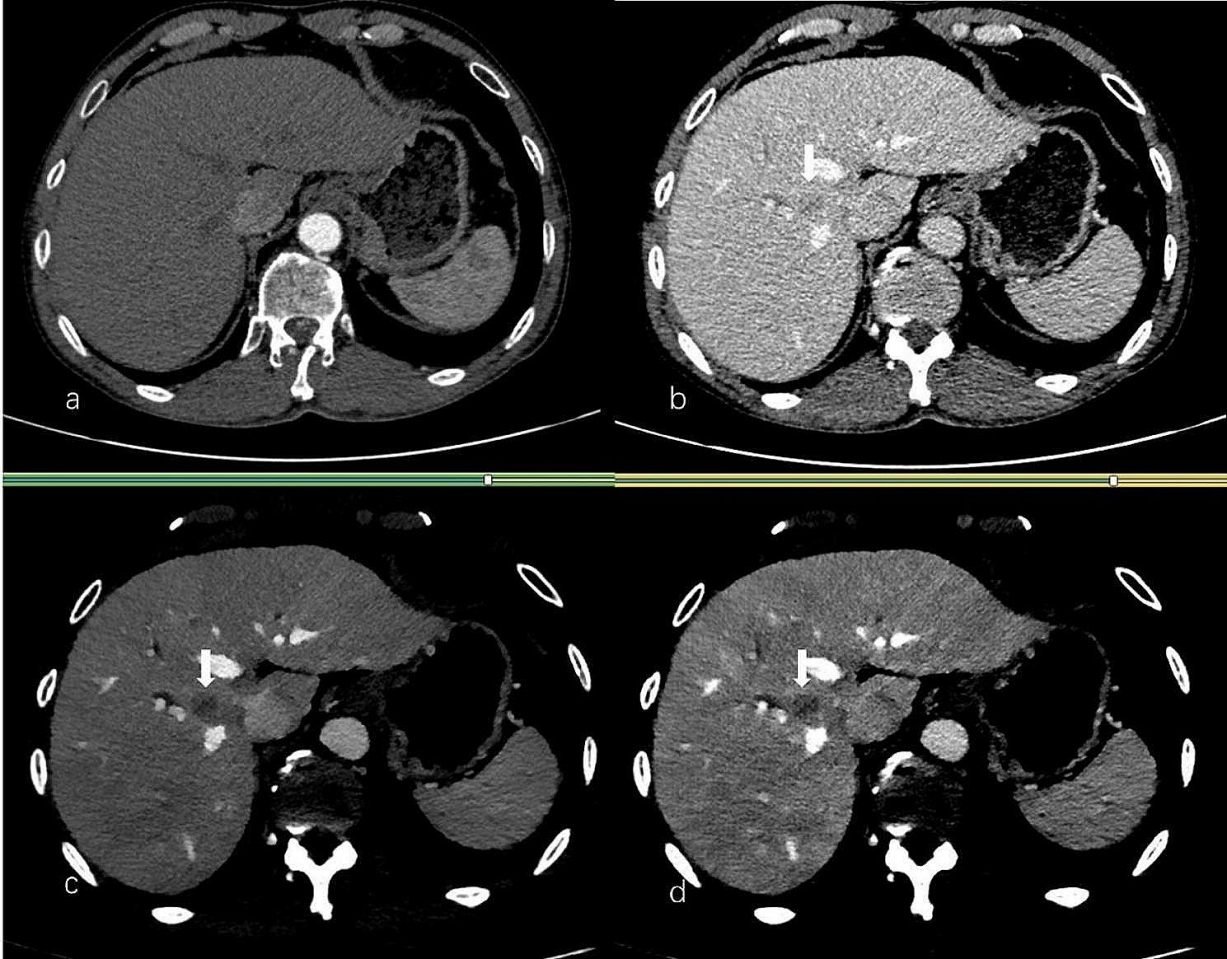
Artery-phase (AP) and portal-vein-phase (PVP) conventional CT image (**a**, **b**); PVP virtual monoenergetic images at 40 keV (**c**); and Gen-VMI_40keV_ (**d**) produced by the best generative model in a patient with HCC (slice thickness of 3 mm). On the conventional AP contrast-enhanced CT images, no lesion was visible (**a**). The lesion was faint in PVP (arrow, **b**). PVP virtual monoenergetic images at 40 keV and Gen-VMI_40keV_ showed HCC, which are more conspicuous (arrow, **c**&**d**)

**Fig. 8:**
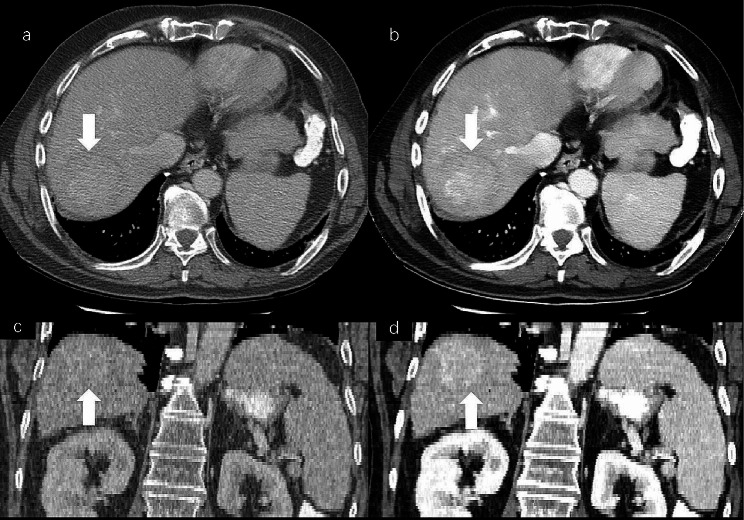
Conventional images of a patients with HCC (**a**&**c**), identified as HCC_019 from the Test group3 (slice thickness of 2.5 mm), Gen-VMI_40keV_ produced by the best generative model (**b**&**d**). On the axial and coronal portal-vein contrast-enhanced conventional images, the lesion was less visible than that on the Gen-VMI40keV (arrow)

HCC is typically identified by its hallmark features such as arterial phase hyperenhancement (wash-in) and hypoenhancement on portal- or delayed-phase images (wash-out) [40, 41]. However, imaging of small HCCs may deviate from the typical pattern due to factors such as well-differentiated HCC, fatty changes, and significant fibrosis within the tumor [42]. Consequently, these variations may complicate the diagnosis of small HCCs by conventional CT. Small HCC do not show portal-phase wash-out at dynamic CT images appearing nearly isodense on conventional images but demonstrate improved delineation on VMI_40keV_ and Gen-VMI_40keV_ (Fig. 7). In this study, the subjective image quality ratings for Test groups 1, 2 and 3 reveal significantly higher image quality for Gen-VMI_40keV_ versus CI. In addition to its application to the data from the three test groups, the best generator model was also applied to other non-spectral CT data. Figure 8 presents a case of HCC in the Test group3, identified as HCC_019.

In the paper, significant structural distortions or artifacts in the Gen-VMI_40keV_ generated by the pix2pix GAN have not been observed. This positive outcome may be attributed to several factors that contributed to the robustness and fidelity of the model. Firstly, the dataset used was comprehensive and contained a substantial amount of data, which likely provided the model with a diverse and representative sample of the imaging task at hand. This extensive dataset would have helped the model to generalize better and avoid overfitting to specific patterns. Additionally, the model’s capacity was carefully chosen to match the complexity of the task, ensuring that it was neither underpowered nor overpowered. The learning rate of 0.0002, which is on the lower end, would have facilitated a more stable and gradual learning process, preventing the model from converging too quickly to suboptimal solutions. The absence of mode collapse, a common issue in GAN training, further indicates that the model was effectively exploring the data space without getting stuck in generating a limited variety of outputs. These factors collectively may have contributed to the high-quality image synthesis observed in the Gen-VMI_40keV_. However, this study focused solely on no apparent abnormalities and HCC of the upper abdomen, and it is possible that other diseases in the upper abdomen may exhibit artifacts or structural distortions.

Image segmentation can divide an image into regions with different semantic information, aiding in the accurate identification of key content within the image, such as the liver, tumors, and other critical areas. These key regions are crucial for image quality assessment because they are typically associated with medical diagnosis and treatment [22, 26]. Additionally, there are other parameters for evaluating image similarity, such as Mean Squared Error (MSE), Normalized Cross-Correlation (NCC), Mutual Information (MI), and the Feature Similarity Index (FSIM) [43]. In future analyses, we plan to incorporate image segmentation to further enhance the specificity of the evaluation. By segmenting the images into different tissue types, we will be able to assess the image quality for each specific region or organ and establish more objective evaluation metrics. This will allow for a more targeted and precise evaluation of the Gen-VMI_40keV_, potentially providing deeper insights into its clinical utility.

In conclusion, this work successfully developed CI-VMI_40keV_ model to generate Gen-VMI_40keV_ from CI and demonstrated its potential clinical utility. Through the evaluation of three test datasets in both objective and subjective aspects, Gen-VMI_40keV_ demonstrated commendable quality comparable to VMI_40keV_ and significantly enhanced the detectability of lesions, reduce the demand for DECT, thus expanding the application scope of advanced imaging technologies, yielding higher diagnostic confidence.

Limitations of this study were: ① only VMI_40keV_ was analyzed, and no relevant analysis was conducted for VMI at different energy levels or for other applications of spectral CT, e.g., iodine and effective atomic number maps. In future research, different spectral images should be expanded to generate spectral images of diverse desired energy levels based on CI; ② only portal venous phase images were analyzed, and future studies should include images from different phases; ③ the validation process was performed specifically on hepatocellular carcinoma (HCC) during testing. It may be beneficial that future studies should incorporate image analysis from a more extensive range of diseases.

## Data Availability

Test group 3 images can be obtained from The Cancer Imaging Archive at https://www.cancerimagingarchive.net/collection/hcc-tace-seg/. The code for the GAN model used in this study is available at https://github.com/picklesdaddy/VMI40kev.

## Abbreviations

VMI_40keV_: Virtual monoenergetic images at 40 keV
Gen-VMI_40keV_: Generative virtual monoenergetic images at 40 keV
CI: Conventional images
GAN: Generative Adversarial Networks
CNN: Convolutional neural network
HCC: Hepatocellular Carcinoma
DECT: Dual-energy CT
MAE: Mean Absolute Error
MSE: Mean Square Error
PSNR: Peak Signal-to-Noise Ratio
SSIM: Structural Similarity
ROI: Region of interest
SD: Standard deviation
SNR: Signal-to-noise ratio
CNR: Contrast-to-noise ratio

## Declarations

### Study subjects or cohorts overlap

No study subjects or cohorts have been previously reported.
